# An analogous wood barrel theory to explain the occurrence of hormesis: A case study of sulfonamides and erythromycin on *Escherichia coli* growth

**DOI:** 10.1371/journal.pone.0181321

**Published:** 2017-07-17

**Authors:** Dali Wang, Zhifen Lin, Ting Wang, Xiruo Ding, Ying Liu

**Affiliations:** 1 State Key Laboratory of Pollution Control and Resource Reuse, College of Environmental Science and Engineering, Tongji University, Shanghai, China; 2 Collaborative Innovation Center for Regional Environmental Quality, Beijing, China; 3 Shanghai Key Lab of Chemical Assessment and Sustainability, Shanghai, China; University of Campinas, BRAZIL

## Abstract

Hormesis has aroused much attention during the past two decades and may have great implications on many fields, including toxicology and risk assessment. However, the observation of hormesis remains challenged under laboratory conditions. To determine favorable conditions under which to observe hormesis, we investigated the hormetic responses of *Escherichia* *coli* (*E*. *coli*) upon exposure of different concentrations of sulfonamides and erythromycin at different time points and in different culture media: Luria-Bertani (LB) broth and Mueller Hinton (MH) broth. Our results reveal that the antibiotics, both individually and combined, produce hormetic effects on *E*. *coli* growth in MH broth at the stationary phase, with the maximum stimulatory response increasing with time. However, in LB broth, the hormetic response was not observed, which can be explained by an analogous “wood barrel theory”. Our study suggests that the culture medium and time should be taken into consideration in hormetic studies, and compound mixtures should also receive more attention for their potential to induce hormesis.

## Introduction

The dose-response relationship is the most fundamental concept in toxicology, and environmental risk assessment is conducted based on this relationship [1]. For a long time, threshold and linear non-threshold (LNT) models have been applied in risk assessment for non-carcinogens and carcinogens, respectively [2]. However, the two traditional models have been challenged with an alternative model that has aroused many concerns over the past two decades [3]. This dose-response model is defined as hormesis, which refers to a biphasic dose-response relationship with low dose stimulation and high dose inhibition [4]. Studies [5,6] have indicated that hormesis can better predict low-dose responses, and the hormetic dose-response model is more common than the threshold model in toxicology. Calabrese [7] has argued that the hormetic dose response relationship should be the default model in risk assessment for non-carcinogens and especially for carcinogens. Therefore, studies on hormesis may have significant implications for the practice of risk assessment.

Currently, hormesis is widely accepted, with numerous hormetic dose response relationships reported in the toxicological literature [8]. It has been stated that hormesis occurs independently of species, endpoint and physical or chemical stressor [9]. Several probable mechanisms have been proposed to account for the low-dose stimulatory responses, such as overcompensation stimulation and receptor mediated stimulation [10]. However, controversy about hormesis remains, the majority of which focuses on whether the phenomenon of hormesis is real because the stimulatory responses are usually modest, which makes it difficult to distinguish hormetic responses from random variation [11]. Therefore, the observation of hormesis remains challenged yet is of great significance in the field.

As discovered by Calabrese [4,7], the observation of hormesis requires a robust study design, adequate statistical power, and reliable replication. With respect to study design, the dose selections, temporal factors and other experimental conditions should be taken into consideration [12]. In particular, the experimental conditions may have important influences on the hormesis occurrence. For example, Vichi et al. [13] reported that the hormetic effects of Adriamycin on cell growth only occurred in partially exhausted medium. Similarly, Belz et al. [14] found that Parthenin induced hormesis only took place at below maximal but still at good growth conditions for root elongation in *L*. *sativa*. In the present study, we investigated the hormetic effects of antibiotics, both individually and combined, on *E*. *coli*. To determine the most favorable conditions for hormesis, diverse culture media and different time points were used. Sulfonamides (SAs) and erythromycin were selected as the study subjects because they are among the most commonly used antibiotics.

The aims of this work are to (1) investigate culture media and time points for their influences on the occurrence of hormesis; (2) determine the hormetic effects on *E*. *coli* growth induced by antibiotics, both individually and combined; and (3) explore the possible mechanisms of the hormetic effects. Our study will provide a valuable reference for the hormesis field.

## Materials and methods

### Chemicals and organisms

All antibiotics were purchased from Sigma-Aldrich Co. LLC. (Shanghai, China), including erythromycin (Ery) and three SAs, namely sulfamethazine (SMZ), sulfadiazine (SD), and sulfameter (SM). The model organism *E*. *coli* MG1655 was obtained from Biovector Co., LTD. (Beijing, China).

### Culture media

Two types of broth media were used in this study: Luria-Bertani (LB) broth and Mueller Hinton (MH) broth. LB broth is composed of 1% tryptone, 0.5% yeast extract, and 1% NaCl with its pH adjusted to 7.0–7.2. MH broth contains 0.3% beef infusion, 1.75% casein hydrolysate, and 0.15% starch with its pH adjusted to 7.2–7.4. The 1-, 1.5-, 2-, and 2.5-fold broth media were prepared through multiplying each of the components by 1, 1.5, 2 and 2.5 times, respectively.

### Toxicity determination

Prior to the toxicity determination, *E*. *coli* was incubated with shaking in 5 mL LB broth medium at 37°C until OD_600_ (the optical density at 600 nm) reached approximately 0.5, which indicated a CFU number of about 6×10^9^/mL (see S1 Fig for more information). Then the inoculum was diluted 1×10^5^ times with 1% NaCl by two steps, *i*.*e*, a 1000-fold dilution followed by a 100-fold dilution. The obtained bacterial dilution contained approximately 6×10^4^ CFU/mL and was used for the toxicity determination. The antibiotics were prepared at a series of concentrations in 1% NaCl (see S1 Table for the concentration design). The solvent DMSO (with a final concentration of 0.5% v/v) was added to the chemical dilution in case the chemicals do not dissolve. The toxicity tests were performed by a Bioscreen C Reader equipped with two honeycomb multiwell plates (Oy Growth Curves Ab Ltd). This instrument consists of an incubator and a measurement unit, which can automatically measure the OD_600_ of each well of the plates during the bacterial incubation. Prior to the incubation, 80 μL chemical solutions, 80 μL broth media, and 40 μL diluted inoculum were successively added to each well of the honeycomb plates. Each well contained a total 200 μL mixed solution. 1-, 1.5-, 2-, and 2.5-fold broth media were added to the wells in order to make final 0.4-, 0.6-, 0.8, and 1.0-fold media, respectively. Each antibiotic concentration was tested in triplicate, and wells with no antibiotics were measured as the controls. DMSO was also added to the control groups with a final concentration of 0.5% v/v. The OD_600_ value of each well was measured at time zero and after exposure for varying periods. For the combined toxicity determination, the binary mixtures were prepared in equitoxic concentrations based on their individual EC_50_ values at 24 h (S1 Table). EC_50_ denotes the median effective concentration—the concentration of a chemical that inhibits 50% of the bacterial OD_600_, which was obtained according to the dose response curves.

### Data analysis

The response of *E*. *coli* to the antibiotics was expressed as %control, which was calculated by dividing the OD_600_ of the exposed groups with the OD_600_ of the controls. It should be noted that the OD_600_ values measured at time zero were subtracted from those made at later time points, correcting for the possible absorbance by tested chemicals at 600 nm. The data analysis was performed on GraphPad Prism software. The response data were reported as mean ± SD according to the results of the triplicates.

The non-hormetic dose-response curves were fitted by the following model:

$$\text{y}=\text{a}+\frac{\text{b}-\text{a}}{1+10^{\left(x-\text{logE}{\text{C}}_{50}\right)}}$$ (1)

where a and b are the bottom and top plateau level of the curve and logEC_50_ denotes the concentration that gives a response half way between bottom and top. The hormetic dose-response curves were fitted by a bell-shaped dose-response model. This model sees the hormetic curve as a sum of two-dose response curves, one that stimulates and one that inhibits. The equation for this model is as below:

$$\text{y}=\text{a}+\frac{\text{b}-\text{a}}{1+10^{({\text{logEC}}_{50,1}-x)\cdot \text{nH}1}}+\frac{\text{c}-\text{a}}{1+10^{(x-{\text{logEC}}_{50,2})\cdot \text{nH}2}}$$ (2)

where a denotes the plateau level in the middle of the curve, b and c are the plateaus at the left and right ends of the curve, logEC_50,1_ and logEC_50,2_ are the concentrations that give half-maximal stimulatory and inhibitory effects, nH1and nH2 are the slope factors.

## Results

### *E*. *coli* growth curves under different media conditions

**Fig 1:**
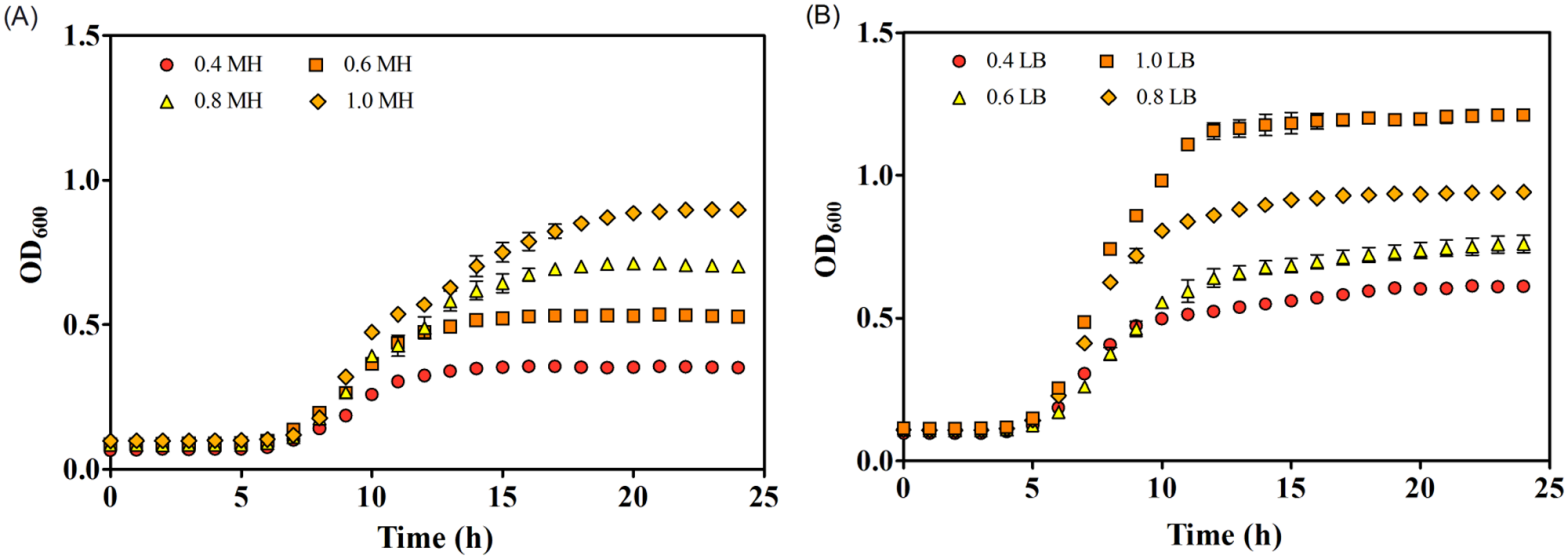
Growth curves of *E*. *coli* in different concentrations of MH (A) and LB broth media (B).

The *E*. *coli* growth curves were investigated in different concentrations of LB and MH broth media. The results (Fig 1) suggested that the LB broth media provided a better condition for *E*. *coli* growth than the MH broth. In general, *E*. *coli* in LB broth showed a shorter lag time, a faster growth rate (at logarithmic phase) and a greater maximum achieved OD_600_ (at stationary phase) than those in the MH media. Moreover, the growth curves of *E*. *coli* also varied with the broth concentrations. As shown in Fig 1, the growth rate and the maximum achieved OD_600_ increased with the broth concentrations, for the cases of both MH (Fig 1A) and LB broth (Fig 1B).

### Effects of antibiotics on *E*. *coli* in different broth media

**Fig 2:**
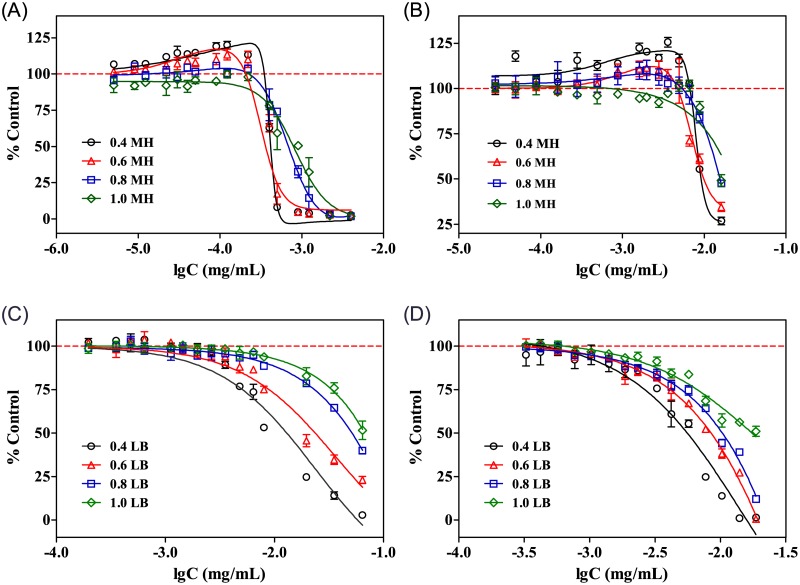
Dose-response curves of antibiotics towards *E*. *coli* in different broth media. (A) SMZ in MH broth; (B) Ery in MH broth; (C) SMZ in LB broth and (D) Ery in LB broth.

The effects of SMZ and Ery on *E*. *coli* were investigated in different broth meida. For each of the broth, 0.4-, 0.6-, 0.8- and 1-fold dilutions were examined. Fig 1 shows the 24 h dose-response curves of SMZ and Ery in MH and LB broth media. According to the results, the hormetic effects of SMZ and Ery on *E*. *coli* could only be observed in 0.4- and 0.6-fold diluted MH broth (Fig 2A and 2B). The differences between the stimulatory groups and the controls were statistically significant according to the one-way analysis of variance (see S2 Fig). In LB broth (Fig 2C and 2D), however, no hormetic effect was observed. It was also found that the magnitude of the hormetic effects in MH broth decreased as the broth concentration increased. For example, in 0.4-fold diluted MH broth (Fig 2B), the maximum stimulatory effect of Ery was up to approximately 125% of the control, which was greater than those in 0.6-fold diluted MH broth (about 15%); while in 1-fold MH broth, no stimulation was observed.

In addition, SMZ and Ery showed higher toxicity to *E*. *coli* in MH broth than in LB broth. For example, the EC_50_ of SMZ in 0.4-fold diluted MH was about 3.98×10^−4^ mg/mL, which was lower than that in 0.4-fold diluted LB broth (2.4×10^−2^ mg/mL). This suggested that *E*. *coli* is much more sensitive to the antibiotics in MH broth than in LB broth. Moreover, the toxic effects also decreased with the broth concentrations for both MH and LB cases, which indicated the bacteria were more sensitive to the antibiotic at lower media concentrations.

### Hormetic effects of antibiotics at different time points

**Fig 3:**
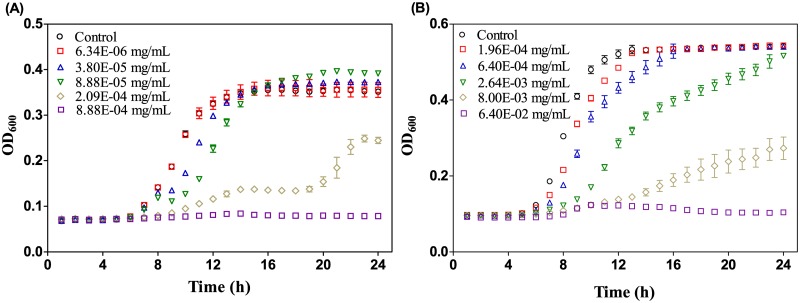
Growth curves of *E*. *coli* exposed to the indicated concentrations of SMZ in 0.4-fold diluted MH (A) and LB (B) broth.

The 0.4-fold diluted MH broth was selected for the subsequent investigation, because the hormetic responses in this broth were the most significant. Fig 3A shows the growth curves (0–24 h) of *E*. *coli* exposed to the indicated concentrations of SMZ in 0.4-fold diluted MH broth. According to the results, 3.80×10^−5^ and 8.88×10^−5^ mg/mL of SMZ induced an increase in the maximum achieved OD_600_ at the stationary phase, which was the cause for the observed hormesis at 24 h in Fig 2A. Interestingly, SMZ with the two concentrations did not show any stimulation on *E*. *coli* at the lag and logarithmic phases. As a comparison, Fig 3B shows the growth curves of *E*. *coli* exposed to SMZ in 0.4-fold diluted LB broth. Unlike the cases with MH (Fig 3A), SMZ in 0.4-fold diluted LB broth did not show any stimulation on *E*. *coli* throughout the whole test period, though it had a similar inhibitory effect at the lag and logarithmic phases.

**Fig 4:**
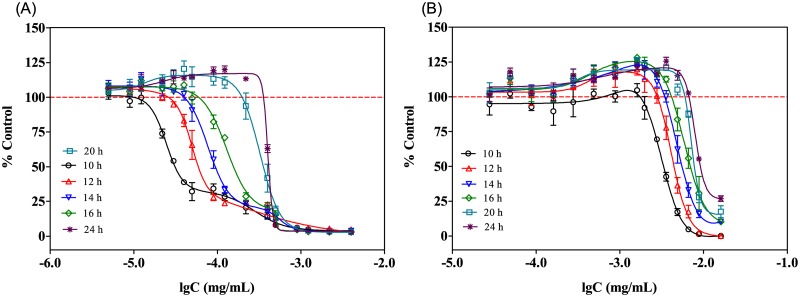
Dose-response curves of (A) SMZ and (B) Ery in 0.4-fold diluted MH broth at different time points.

The dose-response curves of SMZ and Ery in 0.4-fold diluted MH broth were test after different exposure time. According to the results (Fig 4), both the maximum stimulation and the hormetic zones tend to increase with the exposure time. As shown in Fig 3A and 3B, SMZ and Ery within the test concentrations presented no stimulatory effect on *E*.*coli* at 10 h; while after 12 h exposure, SMZ and Ery induced gradually increasing stimulation, which achieved the maximum at 24 h. Moreover, the hormetic zones of SMZ and Ery extended greatly from 10 to 24 h. For example, at 12 h, the hormetic zone (log concentration) for Ery was -3.82 to -2.68, while this extended to -3.82 to -2.31 at 24 h. The dose-response curves of SD and SM at different time points displayed the same changing trends with the exposure time, which were provided in the supporting information (S3 Fig).

### Hormetic effects of binary antibiotics

**Fig 5:**
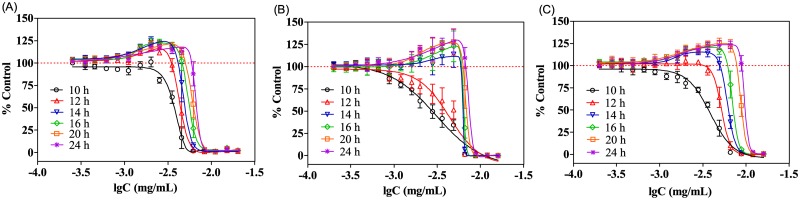
Concentration-response curves for mixtures of SMZ (A), SD (B) and SM (C) with Ery in 0.4-fold diluted MH broth at different time points.

The combined toxicity of SAs and Ery were then determined in 0.4-fold diluted MH broth. The dose-response curves of SAs and Ery mixtures at different times are shown in Fig 5. As is similar to the individual antibiotics, both the inhibitory and stimulatory effects of the binary mixtures were dependent on time. With respect to the inhibitory effects, higher concentrations of the mixtures were needed to provoke the same inhibition at the later time compared to the earlier time. The hormetic effects of the mixtures began to occur at 14 h (Fig 5A) or 16 h (Fig 5B and 5C), which resembles the individual antibiotics (Fig 3). Likewise, both the stimulatory effects and the hormetic zones increased with time, which is also the case with individual SAs or Ery.

**Fig 6:**
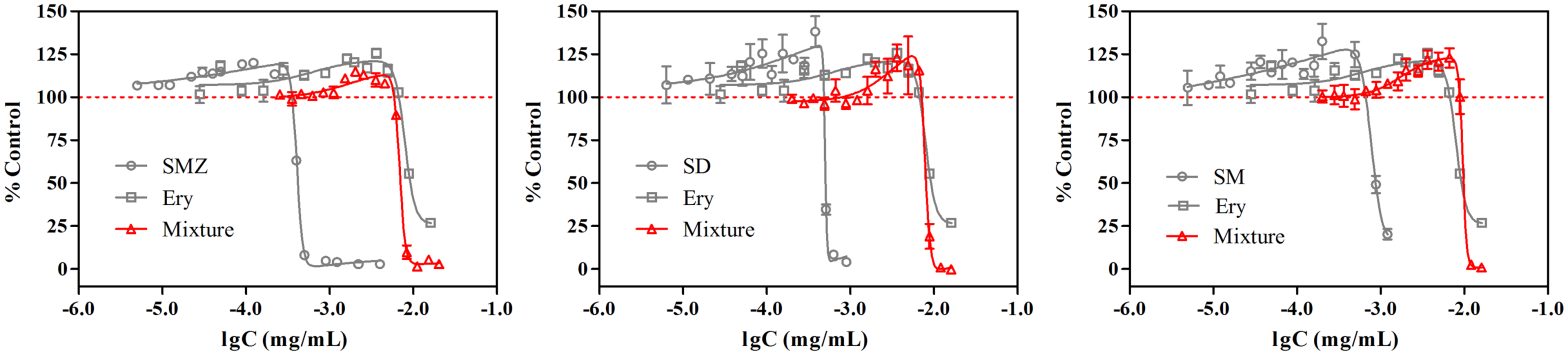
Dose-response curves of SAs, Ery and their mixtures in 0.4-fold diluted MH broth at 24 h.

The comparisons of the individual and combined toxicities of the three SAs and Ery (24 h) were shown in Fig 6. It can be seen that the maximum stimulation caused by the mixture was around 25%, which was comparable to the stimulation caused by the individual components.

## Discussion

### An analogous “wood barrel theory” to explain the absence of hormesis in LB broth

It has been noted by Calabrese et al. [9] that hormesis occurs independently of species, endpoint and physical or chemical stressors. True as it may be, the observation of hormesis is not as easy as we may imagine. Many factors, such as the environmental conditions and exposure time, should be taken into consideration in the study design to observe hormesis [15]. In the present work, the hormetic effects of SAs and Ery on *E*. *coli* were only observed in MH broth (0.4- and 0.6-fold dilutions); while in LB broth media, no hormetic effect could be observed. This result was different from the findings of Calabrese et al.[16], in which the antibiotics in LB broth could induce hormetic effects on the bacterial growth. This was probably due to the different *E*. *coli* strains in the two studies. In Calabrese et al. [16], *E*. *coli* strain MC1601 containing plasmid pET26b (+) was used. This strain has enhanced permeability to chemicals relative to that of the wildtype *E coli* tested in our research.

The comparison of the bacterial growth curves in control and exposed groups (Fig 3) revealed that the hormetic effects could be explained by the greater maximum achieved OD_600_ of the exposed groups than the controls. Additionally, it was found that the maximum achieved OD_600_ of *E*. *coli* differed with the broth types, as well as the broth concentrations (Fig 1). So here comes the question that which factor decides the maximum achieved OD_600_ of *E*. *coli* in a certain culture media?

It was noted that the maximum cell density that the bacteria can achieve in aqueous medium is affected by several factors, such as nutrient supply, oxygen condition, osmotic pressure, pH state, etc. [17]. Therefore, a broth medium can be seen as a wood barrel with boards of irregular lengths, and the cell density in the medium as the water height in the barrel as depicted in Fig 6A. The boards in the wood barrel correspond to the properties of the culture medium, *i*.*e*., the nutrient supply and oxygen conditions. The boards have irregular lengths because these properties may have different influences on bacterial growth, which results in different ceiling densities for the bacteria.

**Fig 7:**
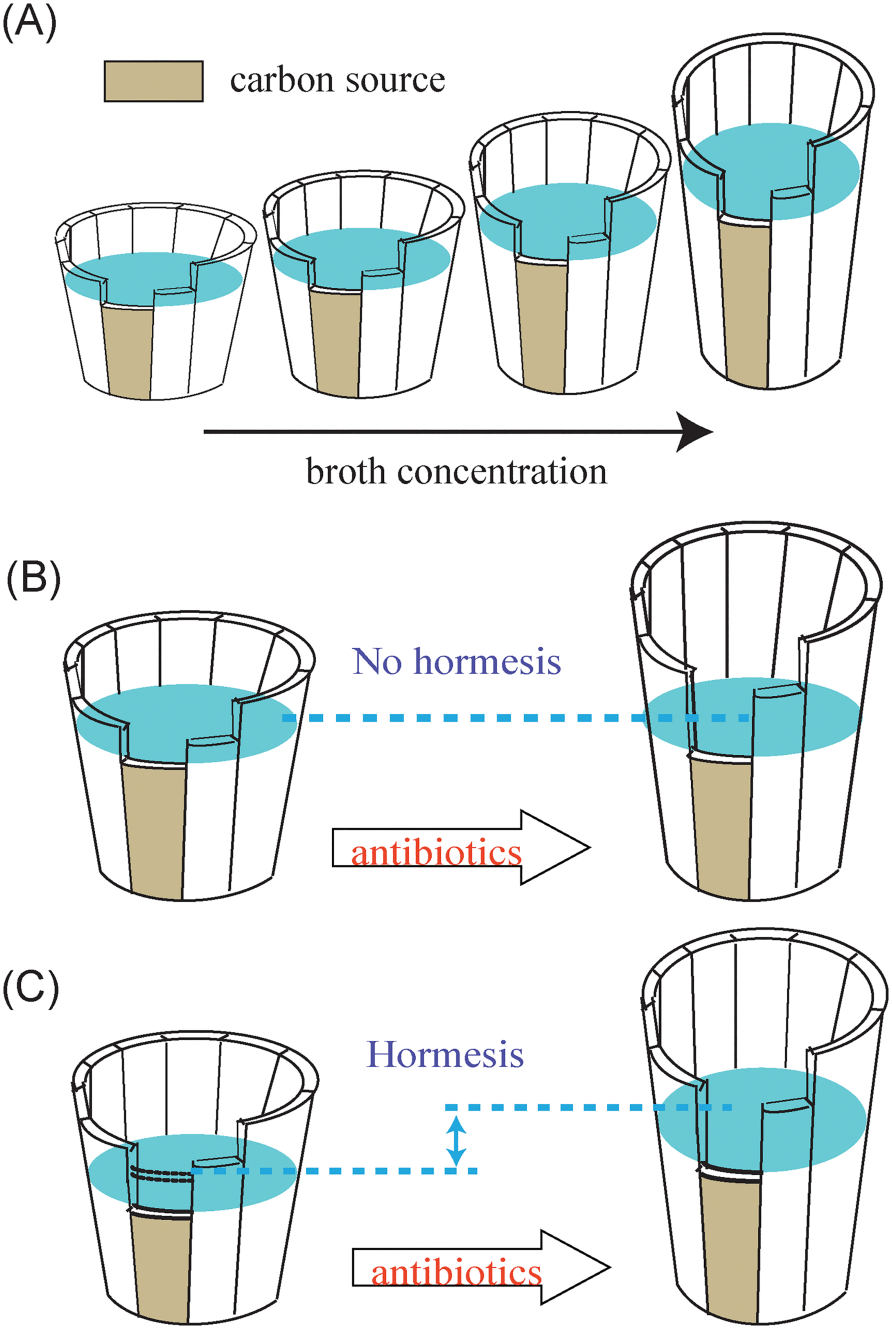
Graphic illustration of the analogous “wood barrel theory”. (A) The culture medium is compared to a wood barrel with irregular lengths, among which the utilizable carbon source is the shortest one. (B) The growth of *E*. *coli* in LB broth was limited by the utilizable carbon source, for which hormesis in LB broth did not show up. (C) *E*. *coli* upon exposure to antibiotics in MH broth was stimulated to make full use of the utilizable carbon source, which leads to a length increase of the shortest board for MH broth, and thus showed a hormetic response.

In our work, the utilizable carbon source in the two broth media was likely to be the shortest board of the wood barrel that limited *E*. *coli* growth (Fig 7A). It was pointed out that LB broth provides small amounts of utilizable carbon sources for *E*. *coli*, though it contains a large total concentration of organic nutrients [18]. As a result, the growth of *E*. *coli* in LB broth usually stops due to a lack of a utilizable carbon source, which is consistent with our findings that the maximum cell density of *E*. *coli* increased with broth concentration, *i*.*e*., the concentrations of the utilizable carbon. In LB broth, we assumed that the antibiotics at low concentrations have the potential to stimulate *E*. *coli* growth through overcompensation or receptor-mediated mechanisms [10]. Thus, there is a possibility that some of the physiological features of *E*. *coli* are stimulated at the individual level by the antibiotics, for example the phenotypic changes [19]. However, from the population level view, the cell density of *E*. *coli* is still limited by the carbon source (the shortest board of the barrel makes no difference after the addition of antibiotics, though the other boards may change, as shown in Fig 7B) and thus cannot exceed the control groups. In other words, the hormetic effects cannot occur at the population level due to the limitation of the utilizable carbon source in LB broth.

With respect to the MH broth, as shown in Fig 3A, the maximum achieved OD_600_ of *E*. *coli* was also dependent on the broth concentration, which suggests that *E*. *coli* growth in MH broth is also limited by the carbon source. However, the case with MH broth may be somewhat different from the case with LB broth. As mentioned above, the maximum cell density in MH broth was smaller than in LB broth. A recent study [20] has shown that MH broth contains a noxious component that is absent from LB broth, and this component is responsible for the growth compromise in this medium. Therefore, when the bacteria reach the maximum cell density in MH broth, the utilizable carbon source may not be used up due to suppression of bacterial growth induced by the noxious component. Upon addition of antibiotics below the toxicological threshold, the suppression of *E*. *coli* growth caused by the noxious component can be eliminated by the antibiotics through some mechanism, e.g., overcompensation or adaptive response. As a result, the bacteria can make full use of the utilizable carbon source in MH broth, which leads to a length increase of the shortest board for MH broth. Consequently, the maximum cell density of *E*. *coli* in MH broth treated with low concentrations of antibiotics can exceed the untreated groups, as shown in Fig 6C. In addition, the concentration of the noxious component in MH broth increases with the broth concentration, which means its suppression of *E*. *coli* growth is enhanced as the MH broth concentration increases. Therefore, in 0.4- and 0.6-fold dilutions of MH broth, the noxious component is at relatively lower concentrations and thus imposes weaker suppression that can be more readily eliminated. As a result, the hormetic effects at lower concentrations of MH broth occur at greater magnitudes.

### Hormetic effects on *E*. *coli* growth are due to an adaptive response

In addition to the culture medium, time is another critical factor that may determine whether hormesis occurs. As stated by Calabrese et al. [8], hormesis may only be observed at certain times after or during exposure because it may be related to an adaptive response. In our study, the hormetic effects of SAs and Ery on *E*. *coli* in MH broth occurred after 14 h at the stationary phase. In the lag and logarithmic phases (before 14 h), SAs and Ery presented inhibitory effects on *E*. *coli* growth in most cases. Smiliarly, Migliore et al. [21] observed that low doses of tetracycline delayed the *E*. *coli* growth at the lag and logarithmic phases, but resulted in an increased OD_600_ value at the stationary phase. Similar results were reported by other researchers [22–25], where chemical or physical stressors caused inhibitory effects in the initial period of observation, after which they evoked stimulatory effects. These dose-time response results suggest that hormetic effects are likely to be the result of an adaptive response that overcorrects in response to the disruption of homeostasis[10]. Therefore, the observed hormetic effects of SAs and Ery on *E*. *coli* growth in the present study were actually due to the adaptive response to inhibition.

### The hormetic effects of mixtures were comparable to those caused by the individual components

In this study, the combination of SAs and Ery was investigated for the hormetic effects on *E*. *coli*. In the real environment, chemicals usually exist as mixtures, so studies on their joint effects on the organism are of great importance. The potential hormetic effects of mixtures have aroused great concerns. Studies have shown that hormesis of individual chemicals does not necessarily lead to hormesis of their mixtures. Binary mixtures with one or both components inducing hormesis could lead to either inhibitory, stimulatory, or no effect [26,27]. In our study, the mixtures of SAs and Ery were observed to induce time-dependent hormetic effects on *E*. *coli* growth. The hormetic effects of the mixtures on *E*. *coli* only occurred at the stationary phase, which again supports the above assumption that hormetic effects on *E*. *coli* are adaptive responses to antibiotic-induced inhibition. The hormetic effects evoked by the mixtures only occurred in MH broth, which was similar to the individual antibiotics.

Within the traditional toxicological framework, the interaction between the components in a mixture is supposed to induce an increased (synergism) or decreased toxicity (antagonism) on the organism or a comparable toxicity (addition). However, within the hormesis framework, the joint effects of the mixture do not infer that there should be an increased or a decreased stimulatory response [28,29]. Fig 6 shows a comparison of the individual and combined toxicities of the three SAs and Ery in which we can find that the maximum stimulation caused by the mixture is comparable to the stimulation of those caused by the individual components, which were all around 25%. The maximum stimulatory response caused by the mixtures was incapable of exceeding the individual antibiotics, suggesting a ceiling density of *E*. *coli* as determined by the utilizable carbon source.

According to the above discussions, SAs and Ery, both individually and in combination, can induce hormetic effects on *E*. *coli* growth at the stationary phase through a probable adaptive response. Our findings suggest that the chemical mixtures should be paid attention to for their potentials to induce hormesis.

## Conclusions

In this paper, we investigated SAs, Ery, and their mixtures for their hormetic effects on *E*. *coli* under various culture conditions and at different times. It was found that hormesis of the antibiotics, both individually and in combination, only occurred in MH broth at the stationary phase, with the maximum stimulatory response increasing with time. The hormetic effects on *E*. *coli* growth were likely due to adaptive effects induced by the antibiotics. The maximum stimulatory response caused by the mixture was comparable to that caused by the individual component. Our study suggests that the culture conditions and time should be taken into consideration in toxicological studies, especially in studies on hormesis, and combination treatments should receive more attention for their potential to induce hormetic effects on organisms.

## Supporting information

S1 Fig Growth curve of *E*. *coli* in 5 mL LB broth medium and the corresponding CFU numbers at 4, 5 and 6 h. (DOCX)

S2 Fig OD_600_ of *E*. *coli* exposed to the indicated concentrations of SMZ and Ery in 0.4- and 0.6-fold MH broth media. (DOCX)

S3 Fig Dose-response curves of SD and SM in 0.4-fold diluted MH broth at different time points. (DOCX)

S1 Table Antibiotic concentrations (mg/mL) used in the toxicity test. (DOCX)

S1 File Raw data. (ZIP)
