# Supplementary material for: An analogous wood barrel theory to explain the occurrence of hormesis: A case study of sulfonamides and erythromycin on Escherichia coli growth
Source: PLoS One. 2017 Jul 17;12(7):e0181321. doi: 10.1371/journal.pone.0181321 (PMC5513561; doi:10.1371/journal.pone.0181321)
Supplement: S1 Fig — (DOCX) [file pone.0181321.s001.docx]

S1 Fig. Growth curve of *E. coli* in 5 mL LB broth medium and the corresponding CFU numbers at 4, 5 and 6 h.

The CFU number was estimated by a pour plate method (*Enclosed Reference*). Specifically, the bacteria were cultured in 5 mL LB medium and the OD_600_ were test by taking 200 μL from the total 5 mL into a 96 well plate at the indicated time points. The bacterial inoculum (at 3, 4, 5 and 6 h) was serially diluted by adding 1x of suspension to 9x of diluent (1% NaCl); a final 10^-8^ solution was made. Then 1 mL of the diluted suspension was inoculated into melted LB agar medium (about 45 °C) and poured in to petri dishes. The dishes were then cultured at 37︒C for 24 h and the colony number on the plates was counted. The number of CFU per ml from the original suspensions was calculated as below:

CFU per ml = Average number of colonies for a dilution × dilution factor (10^8^).

*Enclosed Reference:*

Joseph T. Parisi, Jack N. Baldwin and M. Sottile, Pour-Plate Method for the Detection of Coagulase Production by Staphylococcus aureus. Applied Microbiology, 1973. 25 (4): p. 558-561.
