# Supplementary material for: An analogous wood barrel theory to explain the occurrence of hormesis: A case study of sulfonamides and erythromycin on Escherichia coli growth
Source: PLoS One. 2017 Jul 17;12(7):e0181321. doi: 10.1371/journal.pone.0181321 (PMC5513561; doi:10.1371/journal.pone.0181321)
Supplement: S2 Fig — (DOCX) [file pone.0181321.s002.docx]

S2 Fig. OD_600_ of *E. coli* exposed to the indicated concentrations of SMZ and Ery in 0.4- and 0.6-fold MH broth media. The statistical analyses were performed by Graphpad Prism software. After a Kolmogorov-Smirnov test, a one-way analysis of variance (ANOVA) was used to determine the diﬀerences among treated and control groups, followed by Tukey’s multiple comparison test. Diﬀerences were considered statistically signifcant at p< 0.05, p< 0.01 and p< 0.001, which are labeled with *, ** and ***, respectively.
