# Supplementary material for: An analogous wood barrel theory to explain the occurrence of hormesis: A case study of sulfonamides and erythromycin on Escherichia coli growth
Source: PLoS One. 2017 Jul 17;12(7):e0181321. doi: 10.1371/journal.pone.0181321 (PMC5513561; doi:10.1371/journal.pone.0181321)
Supplement: S3 Fig — (DOCX) [file pone.0181321.s003.docx]

S3 Fig. Dose-response curves of SD and SM in 0.4-fold diluted MH broth at different time points
