# Supplementary material for: An analogous wood barrel theory to explain the occurrence of hormesis: A case study of sulfonamides and erythromycin on Escherichia coli growth
Source: PLoS One. 2017 Jul 17;12(7):e0181321. doi: 10.1371/journal.pone.0181321 (PMC5513561; doi:10.1371/journal.pone.0181321)
Supplement: S1 Table — (DOCX) [file pone.0181321.s004.docx]

S1 Table Antibiotic concentrations (**mg/mL**) used in the toxicity test. The concentration ranges were determined by the preliminary experiments, within with a typical hormetic curve (for hormesis cases) or an S-shpaed curve (for non-hormesis cases) could be observed after 24 h exposure.

| NO. | SMZ | SMZ | Ery | Ery | SD | SM | SMZ&Ery | SD&Ery | SM&Ery |
| --- | --- | --- | --- | --- | --- | --- | --- | --- | --- |
|  | MH | LB | MH | LB | MH | MH | MH | MH | MH |
| EC_50_^a^ | 3.98E-04 | 2.40E-02 | 9.12E-03 | 1.50E-02 | 3.47E-04 | 7.76E-04 |  |  |  |
| #1 | 5.00E-06 | 1.96E-04 | 2.79E-05 | 3.26E-04 | 6.31E-06 | 4.90E-06 | 3.54E-04 | 2.80E-04 | 2.80E-04 |
| #2 | 9.00E-06 | 3.52E-04 | 4.92E-05 | 4.20E-04 | 1.15E-05 | 8.71E-06 | 4.55E-04 | 3.60E-04 | 3.60E-04 |
| #3 | 1.22E-05 | 4.80E-04 | 8.81E-05 | 5.71E-04 | 2.09E-05 | 1.20E-05 | 6.20E-04 | 4.90E-04 | 4.90E-04 |
| #4 | 2.20E-05 | 6.40E-04 | 1.61E-04 | 7.69E-04 | 3.80E-05 | 1.58E-05 | 8.35E-04 | 6.60E-04 | 6.60E-04 |
| #5 | 3.00E-05 | 1.12E-03 | 2.79E-04 | 1.03E-03 | 5.13E-05 | 2.82E-05 | 1.11E-03 | 8.80E-04 | 8.80E-04 |
| #6 | 4.00E-05 | 1.44E-03 | 4.92E-04 | 1.40E-03 | 6.31E-05 | 3.63E-05 | 1.52E-03 | 1.20E-03 | 1.20E-03 |
| #7 | 5.00E-05 | 1.96E-03 | 8.81E-04 | 1.87E-03 | 8.91E-05 | 4.90E-05 | 2.02E-03 | 1.60E-03 | 1.60E-03 |
| #8 | 9.00E-05 | 2.64E-03 | 1.61E-03 | 2.33E-03 | 1.15E-04 | 6.61E-05 | 2.53E-03 | 2.00E-03 | 2.00E-03 |
| #9 | 1.22E-04 | 3.52E-03 | 1.98E-03 | 3.26E-03 | 1.55E-04 | 8.71E-05 | 3.54E-03 | 2.80E-03 | 2.80E-03 |
| #10 | 2.20E-04 | 4.80E-03 | 2.79E-03 | 4.20E-03 | 2.09E-04 | 1.20E-04 | 4.55E-03 | 3.60E-03 | 3.60E-03 |
| #11 | 4.00E-04 | 6.40E-03 | 3.60E-03 | 5.71E-03 | 2.82E-04 | 1.58E-04 | 6.20E-03 | 4.90E-03 | 4.90E-03 |
| #12 | 5.00E-04 | 8.00E-03 | 4.92E-03 | 7.69E-03 | 3.80E-04 | 2.00E-04 | 8.35E-03 | 6.60E-03 | 6.60E-03 |
| #13 | 9.00E-04 | 1.96E-02 | 6.61E-03 | 1.03E-02 | 5.13E-04 | 4.90E-04 | 1.11E-02 | 8.80E-03 | 8.80E-03 |
| #14 | 1.22E-03 | 3.52E-02 | 8.81E-03 | 1.40E-02 | 6.31E-04 | 8.71E-04 | 1.52E-02 | 1.20E-02 | 1.20E-02 |
| #15 | 2.20E-03 | 6.40E-02 | 1.61E-02 | 1.87E-02 | 8.91E-04 | 1.20E-03 | 2.02E-02 | 1.60E-02 | 1.60E-02 |

^a^ EC_50_ in 0.4-fold diluted broth at 24 h. EC_50_ of the single antibiotics were used for the concentration design for the mixtures.
